# Low control beliefs in relation to school dropout and poor health: findings from the SIODO case–control study

**DOI:** 10.1186/1471-2458-14-1237

**Published:** 2014-11-28

**Authors:** Hans Bosma, Marie-José Theunissen, Petra Verdonk, Frans Feron

**Affiliations:** Maastricht University, Social Medicine, CAPHRI, P.O. Box 616, 6200, MD Maastricht, The Netherlands; VU University Medical Center, Medical Humanities, EMGO Institute for Health and Care Research, Amsterdam, The Netherlands

**Keywords:** Health inequalities, School dropout, Control beliefs, Adolescents

## Abstract

**Background:**

There is cumulating evidence that health is compromised through adverse socioeconomic conditions negatively affecting how people think, feel, and behave. Low control beliefs might be a key mechanism. The reversed possibility that low control beliefs might set people on a pathway towards adverse socioeconomic and health-related outcomes is much less examined.

**Methods:**

A case–control design was used, consisting of 330 cases who dropped out of school in the 2010–2011 school year and 330 controls who still attended school at the end of that year. The respondents, aged between 18 and 23, came from Eindhoven and surrounding areas in the south-east of The Netherlands. A questionnaire asked for current health status, recalled socioeconomic and social background, and recalled control beliefs (mastery and general self-efficacy). Logistic regression analyses were used.

**Results:**

Recalls of low mastery and low self-efficacy were strongly related to both dropout and less than good health. Low socioeconomic background was also associated to odds of dropout, but did not confound or moderate the associations of low control beliefs with dropout and health. Odds ratios of dropout and less than good health indicated at least twice the odds of a poor outcome with recalls of low control beliefs.

**Conclusions:**

Independent of the socioeconomic background, low control beliefs are related to heightened odds of both poor health and school dropout. Individual differences in control beliefs might thus be as fundamental as socioeconomic conditions in generating life-course socioeconomic and health-related pathways. Although the findings should first be cross-validated in prospective studies, public health professionals working with youth might already start considering early interventions in youth with all too fatalistic and powerless mind-sets.

## Background

Low control beliefs have often been studied in the context of the higher risks of disease and premature mortality in lower socioeconomic status groups (e.g. [1–4]). Control beliefs can be defined as a person’s belief that he or she is able to actually perform a (desired) action or behaviour (self-efficacy) and the belief that his or her actions matter for outcomes and events (mastery) [5]. Low control beliefs are related to poor health outcomes, through either negatively affecting health behaviours or having direct repercussions for physiological functioning. Some have labelled low control beliefs as powerlessness or “socialised fatalism” [6]. The latter emphasises the embedding in the socioeconomic environment [4, 7]. Low income hampers the number of available options; control over what to purchase is thus restricted. Similarly, long-term low control and autonomy at work, which is common in lower socioeconomic status groups [8, 9], might hinder the development of self-directed behaviours and planning. Low control beliefs have thus been found partially rooted in socioeconomic conditions.

Much less examined is the reversed possibility of low control beliefs negatively affecting socioeconomic attainment (e.g. school dropout) and poor health [2]. High control beliefs might promote social mobility, as people with such beliefs are confident both about their ability to perform well at school and about the future benefits of a higher educational career [10, 11]. The question now is whether control beliefs influence socioeconomic attainment processes and later health and whether this influence is independent of social differences, as indicated by prior socioeconomic and social conditions. An independent influence of control beliefs would suggest that individual differences might be as fundamental as social differences for life-course socioeconomic and health-related pathways. This possibility of selection via individual personality characteristics has insufficiently been examined in the field of socioeconomic differences in health [12].

Using Dutch case–control data on 330 school dropouts and 330 controls still attending school, we set out to examine whether adolescent low control beliefs affect school dropout and poor health in young adulthood. The embedding in the socioeconomic background during upbringing is studied by examining the association of parental socioeconomic and social characteristics during upbringing with control beliefs. Simultaneously, it is examined whether low control beliefs relate to school dropout and poor health, independent of the socioeconomic and social background. The purpose of the study is to help with optimizing interventions aimed at tackling socioeconomic differences in health and to give public health professionals working with youth more personalised tools aimed at a timely prevention of school and health-related difficulties.

## Method

### Study population

This study is part of the Dutch SIODO study, a sequential mixed-methods study, focusing on the early identification of risk factors on the pathway to school dropout [13]. The current paper is using the case–control quantitative data from SIODO. In November 2011, the municipal compulsory education department of the city of Eindhoven selected all young adults aged 18–23 years who had not yet met the Dutch minimum educational requirement, the so-called “basic qualification”, at the start of the 2010–2011 school year. This minimum qualification (for making a good entry on the labour market) is equivalent to higher general secondary education, pre-university education or intermediate vocational education, Level 2. At this age, they normally should have obtained this required qualification. A proportion of these young adults remained in school during the 2010–11 school year to obtain this qualification (the controls), while others dropped out of school during that year without qualification (the cases). Cases and controls being so similar at the start of the 2010–11 school year was supposed to avoid selection bias.

On average 1.5 year after the start of the 2010 – 2011 school year, we sent a self-administered questionnaire with an informed consent form to the eligible young adults. The questionnaire contained questions on the current health status, recalled control beliefs, recalled socioeconomic background, and potential confounders.

The power calculation for a retrospective study with a dichotomous outcome variable indicated that 290 cases and 290 controls would yield an 80% power to detect an odds ratio of 1.75 at a α-level of 5% for an exposure of 0.2 and a ratio of cases to controls of 1 [14, 15]. We had to send 8,630 questionnaires to recruit 1,049 possible participants, among which 330 cases. The 330 controls were randomly selected from the remaining group of participating controls. Approval for conducting this study was granted by the Medical Ethics Committee of Maastricht University (METC 11-4-099, decision 22-08-2011). More detail on the SIODO study can be found elsewhere [13].

### Measures

#### Dropout status and current health status

Dropout status in the school year 2010–2011 was determined by the compulsory education department as defined in the previous paragraph. The subsequent questionnaire asked for the current health status. This was measured by asking how healthy the young adults currently considered themselves (varying from 1: not healthy at all to 10: very healthy). This variable was dichotomised by assigning respondents scoring lower than 7 to the less than good health category (n = 81 (25%) in the case group and n = 32 (10%) in the control group).

### Recalled low control beliefs

For both mastery and general self-efficacy, the respondents were asked to think of the time period when they were 16 (prior to the dropout). Mastery was measured by computing the mean of the seven items of the Pearlin and Schooler scale (0: low and 5: high mastery) (Cronbach’s α = 0.84) [16]. The introduction of this questionnaire asked the respondents to report what was most applicable for them when they were 16. Two example items are: “I have little control over things that happen to me” (to be reverse coded) and “I can do just about anything I really set my mind to do”. General self-efficacy was measured by computing the mean on the 16 items of Sherer’s general self-efficacy scale (0: low and 5: high self-efficacy) (Cronbach’s α = 0.90) [17]. These questions had the same introduction as mastery (asking them to recall their psychological profile at the age of 16). Two example items are: “If something looks too complicated, I will not even bother to try it” (to be reverse coded) and “When I make plans, I am certain I can make them work”. Both variables were categorised into thirds using tertiles.

### Socioeconomic and social background

Socioeconomic background was based on the mean of the standardised scores for educational level of the father and the mother separately and four questions on material and social deprivation. Education had the following ordinal categories: no primary education, primary education only, lower vocational education, intermediate general secondary education, intermediate vocational education, higher general secondary education, higher vocational education, and university education. Separate for the primary and secondary school period, the material deprivation items asked for how often there was too little money for food or for replacing clothes or shoes (never, sometimes, regularly, always). Separate for the primary and secondary school period, the social deprivation items asked for how often there was too little money for joining a (sports) club or going on a school trip (never, sometimes, regularly, always). The resulting composite variable for socioeconomic background was categorised into thirds using tertiles. Ethnic background, looking at both the respondents’ and their parents’ country of birth, was dichotomous, as respondents with a western migration background were too small in numbers (0: autochthonous/western background and 1: non-western background). Family composition during primary school was defined as either living with both parents or living with one parent only.

### Other confounders

Both sex and age at the start of the school year were used as covariates.

### Statistical analyses

First, the associations of the socioeconomic, ethnic, and family background with dropout were analysed using cross-tabulations and related chi^2^-tests. The associations of the background variables with less than good health and low control beliefs were examined in the case and control group, separately. Second, the association of low control beliefs with dropout was also examined by chi^2^-tests. Logistic regression analyses were used to examine the same association, controlling for age and sex, and additionally for the socioeconomic background, ethnic background, and family composition. To examine whether low control beliefs were equally predictive of school dropout in different socioeconomic backgrounds, multiplicative interactions between control beliefs and socioeconomic background were separately tested. Third, the association of low control beliefs with current health was examined by chi^2^-tests in the case and control group, separately. Logistic regression analyses were used to control for age and sex, and additionally for the socioeconomic background, ethnic background, and family composition. Multiplicative interactions between socioeconomic background and low control beliefs were also tested. Sensitivity analyses were done to study the robustness of the findings when using the continuous versions of all variables (and linear regression for current health) and when additionally controlling for the educational level of the first class in secondary school (having four ordinal categories).

## Results

Table 1 shows that the cases, compared to controls, more often came from lower socioeconomic backgrounds (38.2% vs. 28.8%), non-western backgrounds (14.6 vs. 5.2), and one-parent families (21.6 vs. 9.7).

**Table 1:** **Associations of socioeconomic background, ethnic background, family composition and sex with case–control status (column percentages)** ^**a**^

	Controls	Cases
	(n = 330)	(n = 330)
	%	%
Socioeconomic background		*
High	36.4	31.5
Intermediate	34.8	30.3
Low	28.8	38.2
Ethnic background		*
Autochthonous background^b^	94.8	85.4
Non-western background	5.2	14.6
Family composition		*
Two parents	90.3	78.4
One parent	9.7	21.6
Sex		
Women	60.6	60.6
Men	39.4	39.4

*p ≤0.05 (chi^2^-test).

^a^Mean age differed significantly between cases (19.3 years) and controls (19.0 years).

^b^Including a western migration background.

In the case group, low socioeconomic background was related to less than good health (30.4 vs. 17.3) and low mastery recalls (54.8 vs. 32.7) (Table 2). In the control group, adolescents from one-parent families much more often reported low mastery than adolescents from two-parent families (53.1 vs. 22.5). All other associations were statistically not significant.

**Table 2:** **Associations of socioeconomic background, ethnic background, family composition and sex with less than good health, low mastery, and low self-efficacy in cases and controls, separately (row percentages)** ^**a**^

	% less than good health		% low mastery^b^		% low self-efficacy^b^	
	Cases	Controls	Cases	Controls	Cases	Controls
Socioeconomic background	*		*			
High	17.3	10.8	32.7	23.3	36.5	27.5
Intermediate	25.3	6.1	38.0	24.3	37.0	22.6
Low	30.4	12.6	54.8	29.5	47.6	24.2
Ethnic background						
Autochthonous background^c^	25.3	9.6	42.0	25.2	39.9	25.2
Non-western background	21.7	11.8	45.8	29.4	47.9	17.6
Family composition				*		
Two parents	24.9	9.4	39.5	22.5	36.0	25.2
One parent	24.3	12.5	53.5	53.1	59.2	21.9
Sex						
Women	29.3	8.0	45.5	23.5	41.0	26.0
Men	17.7	12.3	38.5	28.5	40.8	23.1

*p ≤0.05 (chi^2^-test).

^a^Mean age differed significantly between those with (19.4 years) and without (18.9 years) less than good health.

^b^Mastery and general self-efficacy were left in three categories, but only the percent in the lowest tertile is shown.

^c^Including a western migration background.

Recalls of low mastery and self-efficacy were strongly related to odds of school dropout (Table 3). For example, adolescents with recalls of low mastery had 144% higher odds of dropout (odds ratio = 2.44) compared with adolescents with high mastery recalls (95% CI: 1.66-3.59). Controlling for the socioeconomic, ethnic, and family background hardly affected these odds ratios.

**Table 3:** **Odds ratios (95% confidence intervals) of school dropout by recalled low control beliefs, adjusted for age, sex (model 1), and additionally adjusted for family composition, and ethnic and socioeconomic background (model 2)**

	Controls (n = 330)	Cases (n = 330)	Model 1	Model 2
	%	%		
Mastery		*		
High	40.0	26.7	1.00	1.00
Medium	34.5	30.6	1.28 (0.87-1.88)	1.27 (0.86-1.88)
Low	25.5	42.7	2.44 (1.66-3.59)	2.15 (1.44-3.20)
Self-efficacy		*		
High	39.4	26.1	1.00	1.00
Medium	35.8	33.0	1.39 (0.95-2.04)	1.30 (0.88-1.92)
Low	24.8	40.9	2.51 (1.70-3.71)	2.24 (1.50-3.35)

*p ≤0.05 (chi^2^-test); percentages are column percentages.

In both the case and control group, the associations of recalled low mastery and low self-efficacy with less than good current health were also substantial (Table 4). After control for the socioeconomic, ethnic, and family composition background, odd ratios indicated a four times higher odds of less than good health for adolescents recalling low control beliefs (e.g. odds ratio = 4.10 (95% CI: 1.48-11.4) for mastery in the control group). The adjustment for socioeconomic, ethnic, and family composition background did not strongly affect the odds ratios (comparing model 1 and 2). Confidence intervals were wide.

**Table 4:** **Odds ratios (95% confidence intervals) of less than good health by recalled low control beliefs, adjusted for age, sex (model 1), and additionally adjusted for family composition, and ethnic and socioeconomic background (model 2) in case and control group, separately**

	Cases	Cases	Cases	Cases	Controls	Controls	Controls	Controls
	N	% less than good health	Model 1	Model 2	N	% less than good health	Model 1	Model 2
Mastery		*				*		
High	88	10.2	1.00	1.00	132	4.5	1.00	1.00
Medium	101	16.8	1.87 (0.78-4.48)	1.81 (0.75-4.36)	114	8.8	1.82 (0.63-5.23)	1.76 (0.61-5.09)
Low	139	39.6	5.92 (2.72-12.8)	5.89 (2.67-13.0)	84	19.0	4.23 (1.56-11.5)	4.10 (1.48-11.4)
Self-efficacy		*				*		
High	86	15.1	1.00	1.00	130	3.8	1.00	1.00
Medium	109	15.6	1.08 (0.49-2.38)	1.09 (0.49-2.43)	118	11.9	3.23 (1.11-9.35)	2.96 (1.01-8.66)
Low	133	38.3	3.68 (1.84-7.38)	3.99 (1.95-8.16)	82	15.9	4.96 (1.67-14.7)	4.78 (1.60-14.3)

*p ≤0.05 (chi^2^-test); percentages are row percentages.

Results of analyses with the original continuous variables, including linear regressions with the health status outcome (Table 5), showed a similar pattern of findings. There were no significant interactions, indicating that recalled low control belief measures are similarly related to poor current health and dropout in all three socioeconomic groups. A similar absence of interactions was detected for ethnic background and family composition. Additional control for the first class level of secondary school that pupils attended did not attenuate the odds ratios for low mastery and low self-efficacy, primarily because of the absence of an association between school level and both mastery and self-efficacy.

**Table 5:** **Unstandardised regression coefficients (95% confidence intervals) of current health status by recalled low control beliefs, adjusted for age, sex (model 1), and additionally adjusted for family composition, and ethnic and socioeconomic background (model 2) in case and control group, separately** ^**a**^

	Cases	Cases	Controls	Controls
	Model 1	Model 2	Model 1	Model 2
Mastery	0.89 (0.67-1.12)	0.83 (0.60-1.07)	0.61 (0.44-0.78)	0.58 (0.41-0.76)
Self-efficacy	0.82 (0.57-1.07)	0.78 (0.52-1.03)	0.58 (0.36-0.79)	0.57 (0.35-0.78)

^a^Ordinary least squares regression analyses using continuous scores of current health status (ranging from 1: poor health to 10: excellent health), mastery (ranging from 1: low mastery to 5: high mastery) self-efficacy (ranging from 1: low self-efficacy to 5: high self-efficacy), and socioeconomic background (continuous score, being the mean of standardised education and deprivation variables).

## Discussion

In this group of 18 to 23 year old Dutch men and women, we found that recalls of low mastery and low self-efficacy were strongly associated with school dropout and less than good health. A lower socioeconomic background, as indicated by measures of recalled relative deprivation and parental education, was also related to school dropout, as was a non-western parental background and coming from a one-parent family. The socioeconomic and social background characteristics did neither confound nor moderate the association of low control beliefs with school dropout and less than good health, which underlines the independent association of low control beliefs with socioeconomic and health-related, life-course pathways.

Some limitations should be discussed. The first two limitations regard the case–control design and the cross-sectional questionnaire. First, we cannot fully exclude the possibility of dropout having affected the low control beliefs (as reported in the posterior questionnaire). Additionally, recall bias could have occurred, when the cases (compared to the controls) had a better remembrance or even reported more prior problems than actually occurred. To avoid these possibilities as much as possible, the questions on low control beliefs were introduced by explicitly asking the respondents to think of the period when they were 16, which was prior to any dropout. Second, the possibility of poor current health affecting the reports of the measures of low control beliefs in the same questionnaire was hopefully also addressed by asking for control beliefs at the age of 16 (and for current health). Dropout affecting the current health status and biasing the estimates of the association between low control beliefs and health was addressed by separate analyses of health status in the case and control group. However, to truly avoid both the first and second limitation, dedicated longitudinal studies are needed that allow a more in-depth and valid unravelling of the causal mechanisms related to socioeconomic attainment and health.

Third, non-response was substantial, as we had to send 8,630 questionnaires to recruit 1049 possible participants (12 percent). The low response rate is related to the sample, and particularly the cases, being a hard-to-reach-group. Non-participation was higher in cases (compared with controls), in boys, and in those living in areas with cheaper houses (Table 6). This may have resulted in an underestimation of the influence of socioeconomic conditions. In the absence of information on differential non-participation according to levels of control beliefs, we do not know whether and how the association of low control beliefs with dropout and health was affected by non-participation. Finally, both cases and controls normally should have had obtained their “basic qualification” already. This increased the similarity in the target population and thus decreased the possibility of selection bias, but might simultaneously have led to underestimated associations of determinants with school dropout. Future studies should examine the associations with a longitudinal design including all possible cases and controls.

**Table 6:** **Participation (in terms of returning questionnaires) according to case–control status, sex, age, and mean house price in postcode of respondent**

	Non-participation	Participation	
	(n = 7581)	(n = 1049)	p-value
Case–control status (%)			<0.001
Controls	92.9	7.1	
Cases/dropouts	79.6	20.4	
Sex (%)			<0.001
Boys	91.3	8.7	
Girls	83.7	16.3	
Age (mean)	19.25	19.09	<0.001
Mean house price (mean)	145690	158380	<0.001

Many studies have conceived of low control beliefs as mediators, rather than as confounders, in the association between low socioeconomic status and poor health [2, 10, 11]. Our findings suggest the importance of thinking beyond “mediation” and of looking at earlier life individual differences (also within socioeconomic groups) as partial driving forces for social mobility, future health, and the development of socioeconomic differences in health in young adulthood. It thus cannot be excluded that control beliefs are as fundamental as socioeconomic grouping when it comes to affecting life-course pathways for people (e.g. see footnote 3 in [18]). Simultaneously, in order to avoid psychologising of social problems, more research is needed to find out where the low control beliefs come from. Environmental factors, possibly other than socioeconomic, might interact with genetic factors in creating these individual differences [12]. Further research could, for example, study how early life parental support, secure attachment and bonding might be important in the development of control beliefs in children and adolescents [19, 20]. Further research should also examine how control beliefs could be more strongly embedded in a personalised approach of public health professionals working with youth (e.g. [21, 22]). Questions in need of an answer are, for example, how to detect low control beliefs, at what age should monitoring start, do gender and childhood diseases matter, how do low control beliefs relate to self-esteem, insecurity and emotional instability (particularly during puberty), and which interventions are available. As reported above, there is, however, first a need for cross-validation of the findings in prospective designs and for more stringent tests of the causal direction of the relevant mechanisms.

## Conclusion

Independent of the socioeconomic background, low control beliefs are related to heightened odds of both poor health and school dropout. Individual differences in control beliefs might thus be as fundamental as socioeconomic conditions in generating life-course socioeconomic and health-related pathways. Although the findings should first be cross-validated in prospective studies, public health professionals working with youth might already start considering early interventions in youth with all too fatalistic and powerless mind-sets.
